# Lipoprotein (a): Underrecognized Risk with a Promising Future

**DOI:** 10.31083/j.rcm2511393

**Published:** 2024-11-06

**Authors:** Matteo Manzato, R. Scott Wright, Allan S. Jaffe, Vlad C. Vasile

**Affiliations:** ^1^Department of Cardiovascular Diseases, Mayo Clinic College of Medicine, Rochester, MN 55905, USA; ^2^Department of Laboratory Medicine and Pathology, Mayo Clinic College of Medicine, Rochester, MN 55905, USA

**Keywords:** lipoprotein (a), ASCVD risk, gene interference therapies

## Abstract

Lipoprotein a (Lp(a)) is a lipid biomarker that binds cholesterol and bears independent cardiovascular risk. Strategies to lower the level of Lp(a) and mitigate such risk are important both for primary and secondary prevention. Currently there are no approved therapies targeting Lp(a) directly. Lipid lowering therapies prescribed routinely may have no effect on Lp(a) levels. Some agents such as niacin and estrogens can significantly decrease Lp(a), but their use is not recommended due to their adverse safety profile. Statins increase Lp(a) levels by 10–20%, questioning the benefit of such therapy when this biomarker is elevated. The Food and Drug Administration (FDA) endorses new agents to address dyslipidemia such as proprotein convertase subtilisin/kexin type 9 inhibitors (PCSK9-i) and Inclisiran, a small interfering RNA. These approaches have been shown to also significantly reduce Lp(a), but more clinical data is needed before implementing their use in clinical practice. Clinical trials are currently ongoing to test the efficacy of newly developed antisense oligonucleotides and small interfering RNAs targeting the gene encoding for Lp(a) in hepatocytes, while other investigations assess small molecules that inhibit Lp(a) assembly. This review summarizes the pathophysiology and clinical implications of Lp(a) elevation, and focuses on proposed Lp(a) therapies and the current state of the clinical trials of such novel agents.

## 1. Introduction

Lipoprotein a (Lp(a)) belongs to the family of lipoproteins which serve as cholesterol transporters. Lp(a) is an LDL-like particle with an apolipoprotein (a) (Apo(a)) covalently bound to apolipoprotein-B (ApoB). The role of Lp(a) in the pathogenesis of atherosclerotic cardiovascular diseases (ASCVD) has been extensively documented in the past two decades [1, 2, 3]. Despite this body of evidence, Lp(a) has not been integrated robustly in cardiovascular risk assessment strategies. Currently, the ASCVD risk calculator in the United States focuses mainly on total cholesterol, high density lipoprotein cholesterol (HDL-c) and low-density lipoprotein cholesterol (LDL-c) values, with great influence placed on age and blood pressure [4]. The Systematic Coronary Risk Evaluation (SCORE) charts in Europe focus on total cholesterol, blood pressure and smoking status. Thus, Lp(a) appears underrepresented in both these risk assessment strategies. This may be particularly important for patients deemed at intermediate risk [5].

The use of niacin and estrogen compounds [6, 7] alter Lp(a) values but none of them provide survival benefit. Statins may increase Lp(a) values, but those changes do not contribute to major adverse cardiac events (MACE) [5, 8]. There is clear evidence that treatment with a proprotein convertase subtilisin/kexin type 9 inhibitor (PCKS9-i) agent will reduce Lp(a) values by 20–25% [9, 10, 11] and provide a modest survival benefit, as evidenced by further analysis of the FOURIER and ODYSSEY OUTCOME trials [12, 13].

There are several ongoing clinical trials evaluating the effects of antisense oligonucleotide (ASO) therapies, small interfering RNAs (siRNA) and small inhibitory molecules to address the unmet need for specific therapies.

The aim of this review is to provide a summary of the biology of Lp(a) and pathological implications of this biomarker and up-to-date data on the latest findings of novel and promising specific approaches to Lp(a)-lowering therapies.

## 2. Lipoprotein a: Structure, Function, and Pathological Mechanisms

Lp(a) and its isoforms are encoded on chromosome 6. The protein component is synthesized mainly in the liver. The mechanisms of Lp(a) assembly are not fully understood. A previous study suggested that Lp(a) was assembled extracellularly, with its ApoB component originating from circulating low density lipoprotein (LDL) [14]. A recent study using the same experimental approach found that ApoB could not come from extracellular LDL because its kinetic properties differed from those of ApoB in LDL [15]. One last hypothesis supports instead a two-step process, whereby the first non-covalent bond is formed intracellularly, while the second disulphide occurs extracellularly [16]. After circulating in the bloodstream, Lp(a) is thought to be cleared similarly to all ApoB containing lipoproteins [17], which involves binding to receptors associated with membrane pits of the hepatocytes’ membrane. The complexes are then internalized and transported to endosomes by clathrin-coated vesicles, where lysosomal-dependent degradation occurs. Renal elimination may also play a role, as evidenced in hemodialyzed patients [18]. Studies on mice suggest very low density lipoprotein (VLDL) receptor — mediated clearance [19], while an *in vitro* experiments highlight the role of megalin, a large glycoprotein also expressed on epithelial tubular cells [20].

**Fig. 1.**
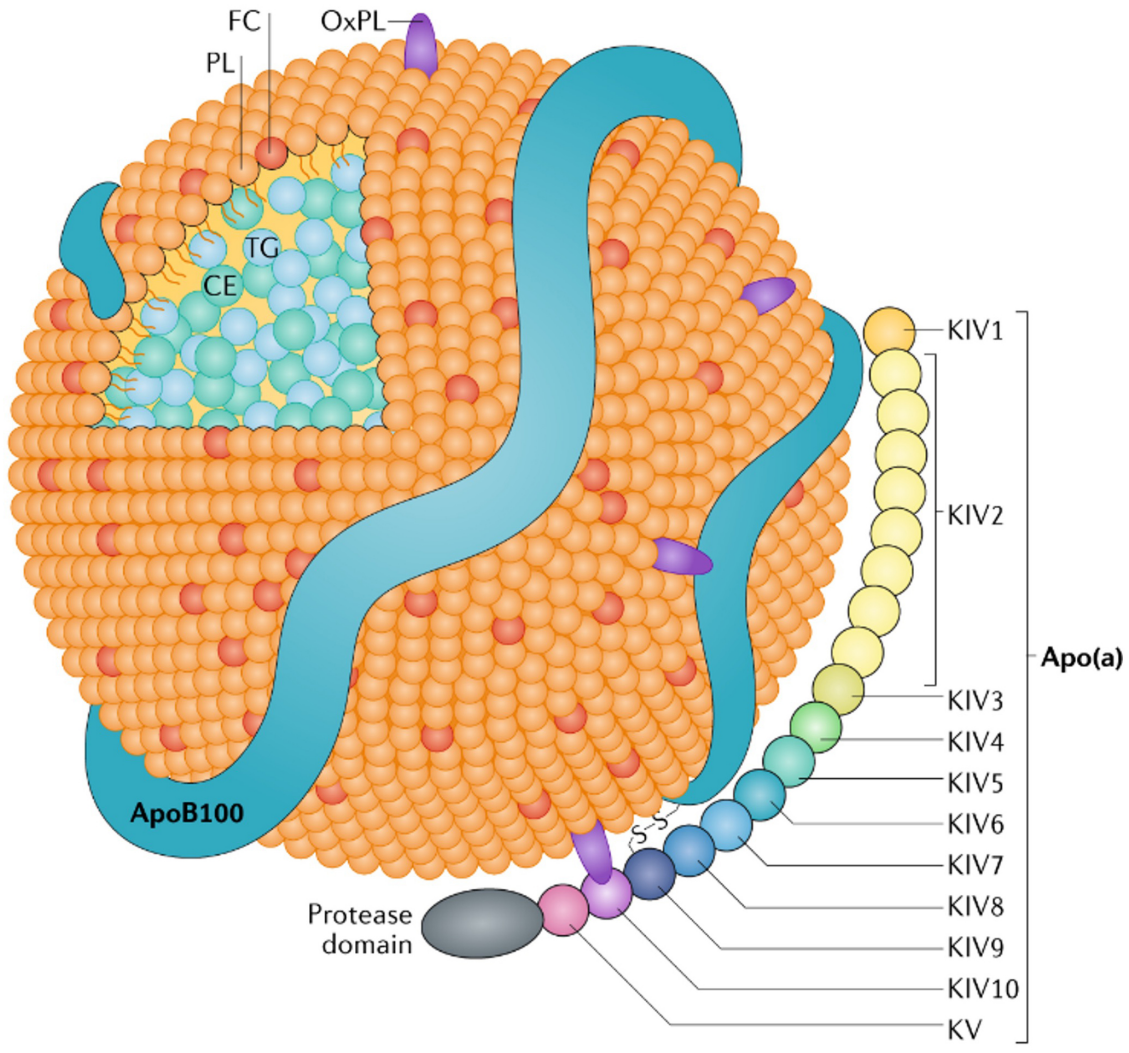
**Lipoprotein a structure**. The major components are the apolipoprotein a (Apo(a)) and the apolipoprotein B-100 (ApoB-100) linked by a disulphide bond. Notice the Kringle domains within Apo(a), which confer Lipoprotein a (Lp(a)) its unique structure. CE, cholesteryl esters; FC, free cholesterol; OxPL, oxidized phospholipids; PL, phospholipids; TG, triglycerides; KIV, kringle IV (fourth - roman) domain.

Lp(a) is an ApoB containing LDL-like molecule with an Apo(a) attached to ApoB-100 by a single covalent disulfide bond [21]. The uniqueness of Lp(a) structure is represented by the “Kringle” protein domain, which consists of 80 amino acids. Variations in Lp(a) levels are highly dependent on the number of copies of the Kringle IV type 2 domain which, along with a Kringle V domain and an inactive protease domain, form the Apo(a) structure [22] (Fig. 1).

The atherogenic potential of Lp(a) can be explained by its increased binding affinity for the endothelial cell wall, inducing expression of vascular cell adhesion molecule 1 (VCAM-1), E-selectin, P-selectin and other adhesion molecules. Further atherogenic properties are conferred by oxidized phospholipids (OxPL), which bind mainly on the Kringle IV10 domain through a lysine bond [23]. As shown in the CASABLANCA study, patients with higher OxPL bound to Lp(a) had higher rates of MACE [24]. In addition to plaque formation, the structure of Lp(a) promotes vascular thrombosis [22] in large part because the molecule bears structural homology with plasminogen. Lp(a) can bind to the plasminogen receptor, inhibiting the action of tissue plasminogen activator and suppressing the fibrinolytic process [22, 25], as demonstrated in *in vitro* studies [26, 27]. Another mechanism proposed for Lp(a) is promotion of inflammation. Along with an increase in reactive oxygen species, Lp(a) triggers the expression of monocytic chemotactic factors, interferon-α and interferon-γ [28]. Moreover, novel molecular pathways have been identified and show that Lp(a) can stimulate M1 macrophage polarization [29]. This data highlight Lp(a) as a strong player between inflammation and atherosclerosis, an important concept in the preventive cardiology space [30, 31].

Circulating Lp(a) concentrations appear to be genetically determined. Hereditability of the *LPA* gene has been shown to be around 90%, [32] suggesting that the likelihood of clustering in first-degree relatives is high. The strongest site of genetic variability is in the Kringle IV type-2 domain. Almost 70% of the Lp(a) coding sequence is located in that region. Indeed, Lp(a) presents around 40 different allelic isoforms encoded by copy number variation (CNV). The molecular weight of these isoforms, based on the number of CNV, is inversely associated with the median concentration of Lp(a) in the blood [33]. Subjects with smaller isoforms manifest higher cholesterol values. This may explain why Lp(a) cholesterol is more predictive of ASCVD than Lp(a) mass [34].

Non-genetic factors may also influence Lp(a) levels, although their contribution is not as well documented, and their role is controversial. Some of these factors include kidney disease, saturated fat intake and steroid hormones [35]. Expression of Lp(a) in different ethnic groups may vary, and atherogenicity of this biomarker in different populations may also differ. Higher plasma concentrations have been reported in Black individuals, whereas Asians seem to have lower mean levels [36]. Moreover, sex differences have also been described, with an increase of Lp(a) in women aged over 50, however no strong difference in cardiovascular outcomes was observed in this population unless Lp(a) levels were greater than 93 mg/dL (199 nmol/L) [37].

**Fig. 2.**
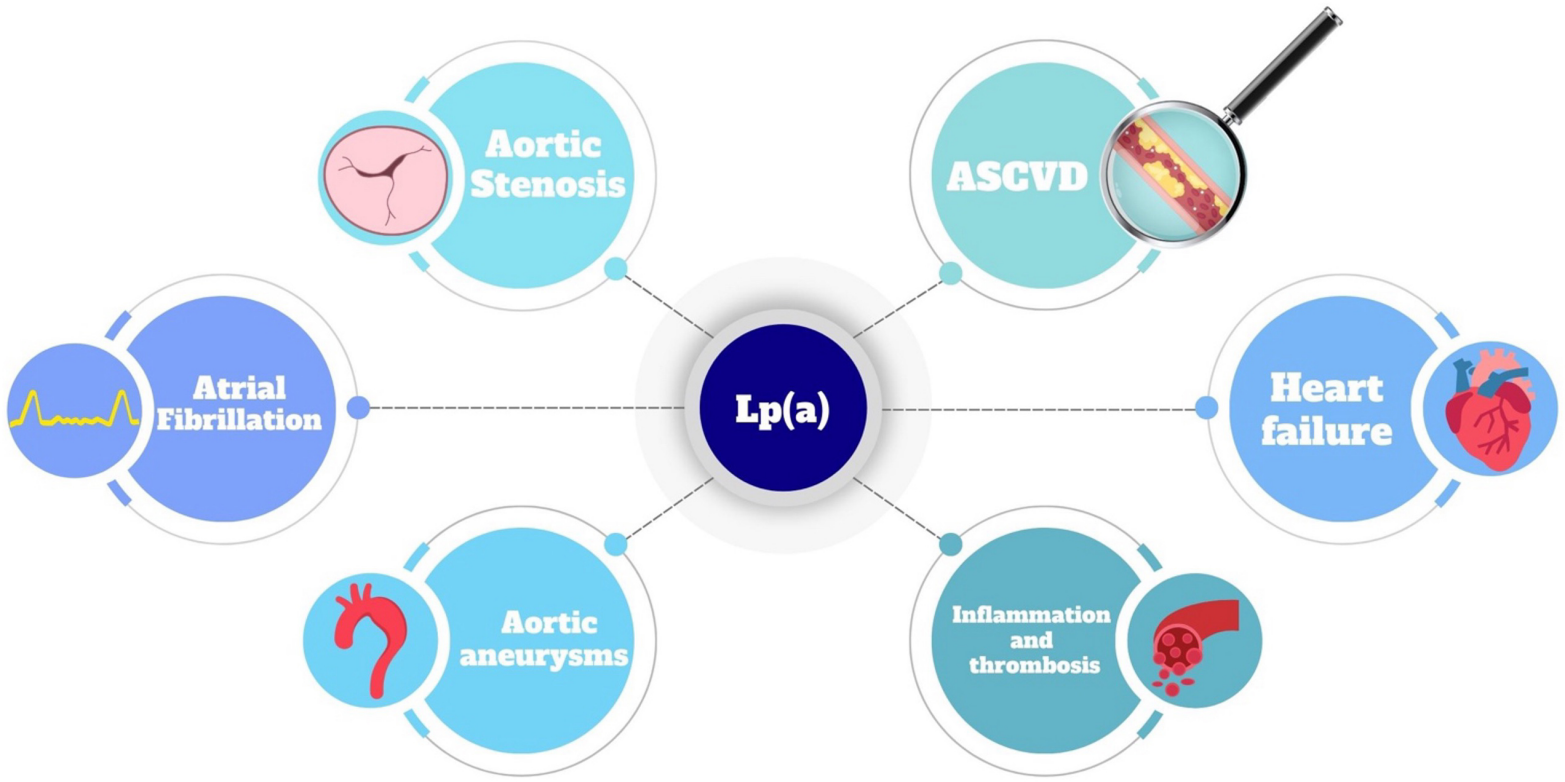
**Lipoprotein a is involved in the onset and progression of several pathologies**. ASCVD, atherosclerotic cardiovascular diseases; Lp(a), lipoprotein a.

To summarize, Lp(a) is associated with several cardiovascular pathologies (Table 1, Ref. [38, 39, 40, 41, 42, 43, 44, 45, 46, 47, 48, 49, 50, 51, 52, 53, 54, 55, 56, 57, 58, 59, 60]; Fig. 2). Lp(a) levels and atherogenicity have a strong genetic component, with only a minor role played by environmental factors. This has important clinical implications for patient screening strategies and may also help identify novel potential therapeutic targets.

**Table 1.** **Pathologies associated with lipoprotein a**.

Condition	Description	References
Atherosclerotic cardiovascular disease (ASCVD)	- Elevated Lp(a) levels are associated with plaque formation and progression.	[38, 39, 40, 41, 42, 43, 44, 45, 46, 47]
	- Residual cardiovascular risk despite LDL-c lowering therapies.	
	- Predisposed subjects with high Lp(a) have higher carotid intima-media thickness.	
	- Dose-response effect: higher Lp(a), higher ASCVD risk.	
	- Patients with CAD and elevated Lp(a) have more ASCVD event recurrences.	
	- Long-term follow-up shows increased residual risk in CAD patients with high Lp(a) levels.	
	- Adding Lp(a) to risk models with LDL-c improves ASCVD prediction accuracy.	
	- Specific *LPA* gene variants increase coronary artery calcification and accelerate necrotic core progression and higher high-risk plaque burden.	
Peripheral arterial disease (PAD)	- Associated with low molecular weight apolipoproteins in symptomatic and asymptomatic patients.	[48, 49]
	- Risk factors like Homocysteine and Fibrinogen levels linked to Lp(a) values.	
Cerebrovascular disease	- Association with ischemic stroke, intracerebral hemorrhage and large artery atherosclerosis.	[50, 51, 52]
Atrial fibrillation	- Proposed mechanism: atrial cellular infiltration creating pro-arrhythmogenic foci, leading to higher mortality.	[53]
Aortic valve stenosis	- Lp(a) promotes aortic valve calcification.	[54, 55, 56]
	- Contrasting evidence regarding association with disease severity and progression.	
Heart failure (reduced ejection fraction)	- Proposed mechanism: maladaptive compensation of damaged myocardium induced by elevated Lp(a).	[57]
Abdominal aortic aneurysm	- Incidence associated with markedly elevated Lp(a) levels.	[58, 59]
	- No dose-response relationship.	
	- Proposed mechanism: inhibition of elastolysis.	
Myocardial fibrosis	- Lp(a) >30 mg/dL associated with significant interstitial myocardial fibrosis, independent of other factors.	[60]
	- Caucasian subjects had higher odds of myocardial scarring.	
	- Increased minimal left atrial indexed volume and decreased left atrial emptying function.	

Lp(a), lipoprotein (a); LDL-c, low density lipoprotein cholesterol; CAD, coronary artery disease.

## 3. Current Guidelines on Lipoprotein a Screening

The 2019 European Society of Cardiology guidelines [61] for the management of dyslipidemias recommend as a class IIa indication that Lp(a) measurement should be considered at least once in each adult person’s lifetime to screen for subjects at increased risk of ASCVD. Moreover, measurement of Lp(a) may be beneficial in patients with family history or premature CVD who are classified as borderline between moderate and high risk. Further consideration of Lp(a) in initiating treatment with PCSK9-i in the setting of elevated Lp(a) is a class IIa indication [61].

On the same note, the 2021 Canadian Cardiovascular Society guidelines [62] for the management of Dyslipidemia strongly recommend measuring Lp(a) once in the lifetime of every person as part of the initial lipid screening. Moreover, more intensive life-style modifications are encouraged for primary prevention if Lp(a) values are >50 mg/dL, whereas initiation of PCSK9-i for secondary prevention may be desirable as elevated Lp(a) levels indicate a strong risk of recurrent events [62].

A different approach has been adopted by the American Heart Association/American College of Cardiology (AHA/ACC). The 2018 Guidelines on the Management of Blood Cholesterol [63] suggest that there is a relative indication to measure Lp(a) for patients with family history of premature ASCVD or personal history of ASCVD not explained by other factors. Thus, Lp(a) is considered as a risk enhancer when higher than 50 mg/dL rather than as part of the comprehensive lipid screening [63]. A recent statement from the National Lipid Association (NLA) instead recommends universal screening for Lp(a) [64]. The authors of this review strongly support the NLA recommendation.

## 4. Laboratory and Clinical Challenges of Lipoprotein a

There are three major controversies when measuring Lp(a): the unit of measurement, the fraction of cholesterol bound to Lp(a), and the cutoff values to identify patients at risk.

Since Lp(a) is encoded variably in the CNV region, its molecular weight can range between 300 kDa to 800 kDa, thus overestimating or underestimating the Lp(a) mass values depending on the size of the calibrator. Molar assays, measuring Lp(a) in nmol/L, reduce both the underestimation due to small Lp(a) isoforms and the overestimation of large Lp(a) isoforms, commonly reported with calibrators in mg/dL [65]. Therefore, molar assays are more supported by the guidelines since they tend to be more accurate [64].

Gel electrophoresis can quantify the density of the Lp(a) band and provide a more accurate estimation of the LDL-c values and the relative contribution of Lp(a) cholesterol contents (Lp(a)-c) to LDL-c. Indeed, if Lp(a)-c is not properly accounted for within LDL-c values, a skewed risk category allocation could occur [66]. By correctly differentiating the Lp(a)-c values contained in the LDL-c component, 3% of the total patient population and 11% of subjects with measurable Lp(a) were reclassified for a diagnosis of familial hypercholesterolemia per the Dutch Lipid Clinic Network criteria. If reclassified with a low score, patients may avoid unnecessary aggressive therapies and/or reflex testing of first-degree relatives [67]. The identification of Lp(a)-c requires more sophisticated laboratory testing which can be performed in highly specialized centers. A less accurate value can be estimated considering that Lp(a)-c corresponds to roughly 30–45% of Lp(a) mass [44].

The last issue is the proper threshold value to define “elevated” Lp(a). Despite 50 mg/dL (125 nmol/L) being widely recognized as the threshold to acknowledge higher risk, a significant portion of the screened population falls within the “gray area” of Lp(a) values in the range 30–50 mg/dL (75–125 nmol/L). These subjects are still at risk for cardiovascular diseases (CVD) and more refined guidelines are needed regarding the management of this subgroup of patients. The current NLA recommendations for Lp(a) management suggest a possible role of repeated testing in these subjects [64].

## 5. Current Therapies Targeting Lipoprotein a

At present, there are limited options for targeting Lp(a), and the majority of therapeutic agents commonly used do not reduce Lp(a) values significantly enough to observe clinical benefits.

Statins represent by far the most employed lipid lowering therapy. Several studies showed a paradoxical increase in levels of Lp(a), ranging from 10.6% to 19.3%, in patients treated with statins [5, 8]. Possible mechanisms behind this finding may be an increased expression of Lp(a) messenger ribonucleic aicd (mRNA) and increased plasma levels of proprotein convertase subtilisin/kexin type 9 (PCSK9) after treatment [68]. It is likely that these changes related to statin therapy do not have any clinical impact since outcome studies have failed to demonstrate an additive negative effect of increased Lp(a) [69, 70].

Estrogen and related compounds have been mainly studied in post-menopausal women and have been found to significantly lower the levels of Lp(a). More specifically tamoxifen [71] and raloxifene [72], two selective estrogen receptor modulators (SERMs) and Tibolone, a synthetic steroid acting as an estrogen substitute, reduce Lp(a) levels in post-menopausal women [7]. The mechanism behind this effect has not yet been elucidated, although an alteration of gene expression and a decreased level of hepatic Lp(a) synthesis have been proposed by several authors [73]. Notwithstanding the promising results, estrogens are no longer recommended due to their intrinsic risk of thrombosis, breast and endometrial cancer.

Niacin reduces Lp(a) with extended-release formulation, independently of treatment duration, dose and percentage change in plasma HDL-c [6]. Niacin has significant side effects such as flushing, rash and gastrointestinal abnormalities [74]. Importantly, it does not reduce the overall incidence of MACE, thus it is no longer recommended despite these effects [75].

Data from Odyssey Outcomes did show an association between lower proportional rates of coronary heart disease (CHD) death and non-fatal myocardial infarction (MI) in those treated with alirocumab and a statin versus a statin alone, across quartiles of Lp(a) values before randomization [76]. Analysis of the FOURIER trial suggested that randomization to a PCSK-9 agent was associated with enhanced reductions in MACE in those with Lp(a) values in the lower quartile but this effect was less evident in those higher Lp(a) [10]. One potential mechanism favoring the concurrent use of statins with PCSK-9 agents focuses on the fact that use of PCSK-9 altering therapy likely reduces cholesterol bound to Lp(a). It is conceivable however that the modest reductions in Lp(a) by PCSK9 agents may be insufficient to offset the risks associated with elevated Lp(a) values. More definitive outcome data with PCSK9 inhibition should be provided with the conclusion of the currently ongoing clinical trials. Some limitations brought forward by PCSK9-i therapies were the compliance of patients due to the parenteral route of administration [77] and the requirement for multiple administrations. Cost and access may also limit the use of PCSK9-i. Currently, PCSK9-i are not approved by the Food and Drug Administration (FDA) to lower Lp(a), although they are endorsed to target LDL-c reduction.

Inclisiran, like PCSK9-i monoclonal antibodies, lowers Lp(a) by 20–25% as demonstrated in Phase 3 clinical trials [78]. The LDL-c lowering efficacy of Inclisiran is not impacted by elevations in Lp(a) and presumably the reductions in Lp(a) will be associated with reduced CV risks, as has been demonstrated in the FOURIER [12] and Odyssey Outcomes [13] trials where Lp(a) was examined. To date, there has not been a summary evaluation examining Inclisiran specifically with regard to outcomes across Lp(a) levels as the three ongoing outcome trials with Inclisiran have not completed.

Apheresis is only considered clinically in severe cases with a very high cardiovascular risk and worrisome Lp(a) levels. Regular apheresis may reduce Lp(a) of 60–70%, with a mean interval concentration of 25–40%, although the practicality of the approach is highly debated [79]. Given that removal of circulating Lp(a) is the main mechanism, rapid return to baseline levels is expected. Moreover, frequent side effects are observed, including nausea, flushing, anemia and hypotension. These may significantly aggravate ischemia in patients with severe atherosclerotic disease.

To summarize, the current state of the art does not offer robust solutions for the reduction of the cardiovascular risk excess conferred by elevated Lp(a). For this reason, novel therapies based on antisense oligonucleotides, small interfering RNAs and small molecules strategies have emerged in recent years.

## 6. Novel Therapies for Lp(a)

### 6.1 Antisense Oligonucleotides

Antisense oligonucleotides modulate gene expression and protein production by binding highly specific sequences on the target RNA. ASO are single stranded DNA fragments, 15–20 nucleotides in length, complementary to an mRNA sequence. More specifically, a central sequence of 8–10 deoxy-ribonucleotides is flanked by ribonucleotides, which enhance the affinity for the target necessary to avoid any off-target effects [80]. Indeed, even a difference of 6–7 base pairs may cause off-target effects [81]. If the ASO correctly binds to the target mRNA, interference can occur through different pathways. The newly formed heteroduplex acts as substrate for the endonuclease Ribonuclease H1 (RNase H1), leading to cleavage of the mRNA and a subsequent decrease in translation levels [82]. ASO can alternatively act as steric hinders, preventing the interaction between mRNA and the 40S subunit of the ribosome, a key component in protein translation. The efficacy of this last mechanism of action is strictly related to the affinity of the ASO for the mRNA [83].

A further way of modulating gene expression is related to the ability of generating alternative splicing by binding to precursor mRNA (pre-mRNA) and either preventing the access of spliceosome to transcript sites, or by correcting an already present mutation, creating a short but functional protein, which today is a widely used method in neurological diseases [84].

In recent years, several ASO have been developed to target different components of the lipogenic pathway, such as mipomersen, targeting the *ApoB* gene, but also other compounds targeting the *PCSK9* gene and ApoC-III [85]. Among these agents, the novel ASO Pelacarsen specifically targets the *LPA* gene.

#### Pelacarsen

Pelacarsen is an ASO that, after entering hepatocytes, interferes with *LPA* gene expression by RNase H1 mediated cleavage. Thanks to chemical stabilization through substitution of oxygen atoms with sulfur on the internucleotide phosphates, the ASO is resistant to degradation and can exert prolonged effects by binding to other Apo(a)-coding mRNAs, further preventing their translation. The current version of Pelacarsen (APO(a)-LRx) utilizes a N-Acetylgalactosamine (GalNac) moiety to facilitate hepatic uptake of the agent. The GalNac ligand increases affinity for hepatic tissue, and promotes internalization of ASO into hepatocytes.

It has been shown that, at much lower doses, APO(a)-LRx reduces Lp(a) levels up to more than 90% compared to the non-conjugated compound [86].

In a phase 1clinical trial conducted by Tsimikas *et al*. [87], a single subcutaneous injection of APO(a)-Rx for the single-dose part of the study, or six subcutaneous injections, during a 4-week period, for the multi-dose part, were randomly given to healthy subjects with Lp(a) values >100 mg/L.

The primary efficacy endpoint was percentage change of Lp(a) at 30 days or 36 days for the single dose or multiple dose cohorts, respectively.

Progressive percentage reduction was noticed with increasing doses of APO(a)-Rx, reaching a maximum reduction of 77.8% in the 300 mg group. The nadir of Lp(a) was reached on day 36. Side effects were only mild, and the medication was well tolerated [87].

A subsequent phase II clinical trial [86] utilized an escalating dose approach. APO(a)-Rx was given once a week for 4 weeks at doses of 100 mg, 200 mg, and then 300 mg. The primary endpoints were percentage reduction of Lp(a) at 85 or 99 days. A reduction of 66.8%–71.6% in Lp(a) levels was observed. The major adverse events reported were influenza—like reactions and injection site reactions.

LDL-c levels were decreased along with ApoB. This finding may be explained by a decrease competition for the LDL receptor binding on hepatocytes secondary to lower levels of Lp(a) after Pelacarsen administration. Moreover, LDL-ApoB-100 particles deprived of the Lp(a) component may be cleared faster, showing a concomitant reduction in LDL-c levels [88].

No serious adverse event, nor injection site reactions were observed in APO(a)-LRx treated subjects, further shifting the attention toward the conjugated compound. A more recent phase II clinical trial, again testing APO(a)-LRx, showed efficacy and tolerability also in subjects with established CVD. Similarly to the previous investigation, decreased levels of LDL-c were observed [89].

To summarize, one of the major advantages of Pelacarsen is the ability to lower Lp(a) irrespective of the isoform encoded [90]. Moreover, the unique design of the phase II trial [89], which consists of the calculation of LDL-c corrected for Lp(a) levels, allows to estimate the effect of the ASO on LDL-c more precisely. Pelacarsen had modest effects on LDL-c, comparable to low dose statins or ezetimibe [86].

We believe that clarification of the role of Lp(a)-c in the reduction of LDL-c should be encouraged in future clinical trials for all the compounds being tested.

Compared to siRNAs, Pelacarsen requires more frequent administrations and reduction of Lp(a) is marginally lower, despite still being largely significant.

Currently, two phase III clinical trials are reported on clinicaltrials.gov. The LP(a) HORIZON trial (NCT04023552) aims at demonstrating a reduced risk of expanded MACE, whereas the second aims to demonstrate the decrease necessity of apheresis in subjects treated with APO(a)-LRx (NCT05305664).

### 6.2 Small Interfering RNAs

Small interfering RNAs are double stranded RNAs (dsRNA) able to regulate gene transcription by interfering with mRNA processing and protein translation through their actions in the RNA silencing complex. Initially described by Fire* et al*. [91] in 1998 in plants and subsequently in animals, very few molecules of dsRNA were necessary in order to alter gene expression.

The dsRNA that initiates the interference pattern for Lp(a) is first cleaved into 21–23 nucleotide fragments named siRNA, which are subsequently loaded onto a pre-RNA-induced silencing complex (pre-RISC), responsible for the cleavage and removal of one of the two strands of the siRNA [92]. The now single-stranded siRNA is incorporated in the RISC protein complex along with a protein of the Argonaute family, which guides the siRNA and can therefore bind to a complementary sequence of target RNA. Complex-mediated cleavage of the target subsequently occurs, thereby reducing gene expression [93].

Along with the FDA approved Inclisiran, three main molecules are currently under investigation.

#### 6.2.1 Inclisiran

Inclisiran is a double stranded siRNA conjugated to a GalNac ligand that inhibits PCSK9 synthesis by hepatocytes. Inhibition of PCSK-9 synthesis through the RNA-induced silencing complex lowers PCSK9 production, which then leads to increased surface expression and durability of pre-existing LDL receptors on hepatocytes, and lowers plasma LDL-c by increasing hepatic clearance of plasma LDL-c.

Pooled phase III data by Wright *et al*. [94] demonstrated a 26.5% reduction in Lp(a) values compared to placebo changes across the ORION 9-10-11 trials.

Inclisiran is now FDA- and European Medicines Agency (EMA) approved for use in patients with heterozygous familial hypercholesterolemia or patients with past history of CVD who are resistant or intolerant to statins. The administration regimen is 284 mg initially and after three months, followed by injection every 6 months. This brings a net advantage to Inclisiran compared to monoclonal antibodies, given that the latter have to be administered every 2–4 weeks.

It is important to underline that the primary target of Inclisiran is reduction of LDL-c, with a favorable effect also on Lp(a). Thereby, this approach may be desirable in subjects with elevated levels of both markers.

#### 6.2.2 Olpasiran

Olpasiran is a siRNA altering the expression of the *LPA* gene by degradation of the mRNA encoding for Apo(a). The target organ is the liver, mediated by GalNAc binding to hepatocytic lectins, more specifically asialoglycoprotein. Once transported inside the hepatocytes, Olpasiran acts accordingly to the mechanism for siRNAs described above [95].

In January 2022, Koren *et al*. [96] set the bases for further research with the publication of preclinical development and a phase 1 clinical trial for Olpasiran. In this study, seven cohorts of patients were recruited.

Subjects were followed for 225 days. Lp(a) reduction showed dose-dependent behavior with a reduction ranging from 70% to more than 90%. Although Lp(a) values increased after reaching nadir between 43rd and 71st day, a significant decrease compared to placebo was still observed at day 225.

Of note compared to placebo, there was a minimal effect on LDL-c and ApoB and no difference in HDL-c and triglycerides levels were observed. No significant drug-related adverse events were noted [96].

In September 2022 O’Donoghue and colleagues [97] published the OCEAN(a)-DOSE trial study design. It was a phase II multicenter randomized double blind, placebo-controlled clinical trial.

281 subjects with Lp(a) >150 nmol/L (60 mg/dL) and a known history of ASCVD were enrolled. Patients were excluded if they had chronic kidney disease (CKD) stage 4 or greater, acute liver disease, New York Heart Association Heart Failure (NYHA HF) class III or IV or an ejection fraction less than 30%, MACE in the previous 6 months or planned revascularization.

Individuals were randomized to four different doses of Olpasiran of 10, 75 or 225 every 12 weeks, and 225 mg every 24 weeks, or a placebo every 12 weeks.

The follow-up lasted 48 weeks with extended safety monitoring for at least 40 weeks thereafter. At baseline 88.3% were on statins, 52% on ezetimibe and 23.1% on PCSK9-i [95].

The primary endpoint was the percent reduction in Lp(a) from baseline level at 36 weeks, whereas secondary endpoints included Lp(a) reduction at 48 weeks along with LDL-c and ApoB reduction at 36 and 48 weeks.

From a median baseline concentration of 260.3 nmol/L, the placebo adjusted mean reductions of Lp(a) were as follows: 70.5% with the 10-mg dose, 97.4% with the 75-mg dose, 101.1% with the 225-mg dose administered every 12 weeks, and 100.5% with the 225-mg dose administered every 24 weeks. All the dosages were found to statistically significantly reduce Lp(a) levels.

Olpasiran has moderate effects on LDL-c levels, showing a placebo adjusted percent reduction of 23.7%, 22,6%, 23.1% and 24.8% for the treatment arms mentioned above, respectively.

The reduction in Lp(a) was persistent at 48 weeks with only minor, non-significant variations, except in the arm treated with 225 mg every 24 weeks, hinting toward a possible administration regimen every 12 weeks. Pain at the injection site was the main adverse event reported, and overall similar events were noted across all the groups [97].

To summarize, Olpasiran has high selectivity for Lp(a), with only modest effects on LDL-c reported in phase II trial but not in phase I. An Olpasiran-based regimen may be appropriate for subjects with high risk ASCVD mainly attributable to elevated Lp(a).

Early screening for elevated Lp(a) of young adults with normal LDL-c may identify a category of patients who would benefit from Olpasiran as a possible preventive strategy to lower the risk of future cardiovascular complications. To date, no clinical trial for primary preventive end points has been carried out.

A phase III clinical trial, (OCEAN(a)) - Outcomes Trial, is registered on (NCT05581303) and the estimated completion date is set to December 2026.

The primary outcome aims are CHD death, MI and urgent coronary revascularization. Notable exclusion criteria include severe renal dysfunction, severe heart failure, hepatic dysfunction and planned cardiac surgery or arterial revascularization.

#### 6.2.3 Zerlasiran (SLN360)

Zerlasiran is a double stranded siRNA coupled to a GalNAc moiety by a chemical linker. The molecule works by degrading mRNA post transcription, preventing translation. The target organ is once again the liver, with increased affinity thanks to the GalNAc moiety [98].

In May 2022 Rider *et al*. [98] reported the pre-clinical assessment of Zerlasiran. Strong effects on *LPA* gene suppression were observed *in vitro*, both on *cynomolgus* and human hepatocytes, with a reduction of up to 95% of Lp(a) values lasting more than 9 weeks. Interestingly, no effect on the *APOB* gene was observed, indicating the high specificity of Zerlasiran.

A single subcutaneous dose of either 3 or 9 mg/kg were given to female *cynomolgus monkeys*. The animals, assessed after two weeks, showed an 85% and 95% reduction of Lp(a) levels in the two groups, respectively. After 9 weeks, Lp(a) levels increased but still maintained a significant reduction compared to baseline (50% and 88% respectively) [98].

Further preclinical toxicological studies revealed no genotoxicity. There was no cardiovascular toxicity, assessed *in vitro* by human ether-a-go-go-related gene *hERG*, and *in vivo* by electrocardiogram (ECG). Thrombocytopenia, kidney and liver toxicity were tested, given the previously reported toxicities with antisense oligonucleotides used in other conditions [99], but no evidence of them was reported [100].

Nissen and colleagues [101] reported the phase 1 APOLLO trial results. A single ascending dose of Zerlasiran was given to 24 out of the 32 participants. The other 8 individuals were assigned to placebo. Individuals had Lp(a) values greater than 150 nmol/L (60 mg/dL) and no history of CVD. Tested doses were of 30, 100, 300 or 600 mg administered subcutaneously.

The primary end points were safety and tolerability of Zerlasiran. The secondary endpoint was a change in levels of Lp(a) after 150 days. In addition, inflammatory markers and changes in ApoB levels were monitored.

Zerlasiran was safe given there were primarily only grade 1 injection site reactions reported. Of note, 4 out of 6 participants in the 600 mg arm reported a grade 2 reaction.

Considering secondary outcomes, the median percentage reductions were 10% for the placebo group, and 46%, 86%, 96% and 98%, for the 30-mg, 100-mg, 300-mg, and 600-mg Zerlasiran groups, respectively, at 150 days of follow-up.

As noted with Olpasiran, Lp(a) concentration progressively increased, but never reached baseline values.

An initial decrease of LDL-c between 13% and 26% was noted at day 30, but the LDL-c concentration returned to baseline levels at day 150, except for the 600 mg group, in which a mild reduction of 10% was persistent. Despite no statistical significance being provided, it can be inferred that no significant changes in LDL-c were observed. Moreover, a dose-dependent increase of C-reactive protein (CRP) was observed during the first week, rapidly trending down thereafter [101]. Overall, no significant toxicities were noted, and a net positive effect on Lp(a) reduction was observed in this study.

A recent update on the follow-up at 365 days in groups receiving higher doses of the compound showed persistent Lp(a) suppression in the range of 45–50%, and no toxicities were reported during the follow up period [102].

At present, a phase II clinical trial is registered on clinicaltrials.gov (NCT05537571) and the study completion date is set for November 2024. The trial is evaluating subjects with elevated Lp(a) and high cardiovascular risk.

#### 6.2.4 Lepodisiran

The last and more recent siRNA being investigated is Lepodisiran. Three trials are registered on clinicaltrials.gov. Two of them are phase 1 trials aiming at assessing dosage in healthy participants (NCT04914546) and in subjects with impaired renal function (NCT05841277).

The third (NCT05565742) is a phase II randomized, double-blind placebo-controlled study aimed at investigating efficacy and safety of Lepodisiran in adults with elevated Lp(a). The study completion date is set for October 2024.

During the AHA 2023 annual meeting in Philadelphia, Nissen and colleagues [103] presented the results of the first phase 1 clinical trial cited.

A single ascending dose trial recruited 48 patients without CVD and Lp(a) >75 nmol/L. Participants were administered doses of 4 mg, 12 mg, 32 mg, 96 mg, 304 mg or 608 mg, or placebo. A progressively stronger Lp(a) reduction was observed with escalating doses, reaching 96% in the 304 mg group and 97% in the 608 mg group. A long lasting effect of this medication was observed, with persistence of Lp(a) lowering 48 weeks after Lepodisiran administration. The drug was safe, showing only mild injection site reactions [103]. Like other siRNAs, the GalNac moiety in Lepodisiran facilitates a high affinity for hepatocytes, binding to the asialoglycoprotein receptor. Notably, Lepodisiran exhibits a distinctive structure compared to other drugs discussed in this review, featuring a hairpin loop. Additionally, chemical modifications confer resistance to degradation by ribonucleases explaining the prolonged effect on Lp(a).

What sets this compound apart is its novel characteristic of providing a long-lasting effect for 48 weeks, distinguishing it from other compounds in the same category, in which Lp(a) levels started to slowly rise again.

The major advantage is that Lepodisiran may only necessitate yearly injections, aligning conveniently with annual follow-ups. While these initial findings are reassuring, the full extent of Lepodisiran’s impact on the prevention of adverse outcomes will be better understood through the upcoming phase II and III trials, given that the subjects enrolled in the phase 1 trial were healthy individuals with no history of CVD [103]. The phase II clinical trial registered on clinicaltrials.gov (NCT05565742), is currently enrolling patients with Lp(a) levels >175 nmol/L.

### 6.3 Small Molecule Inhibitor Therapy

#### Muvalaplin

One novel compound recently developed is Muvalaplin, a small molecule which inhibits the initial binding between Apo(a) and ApoB-100, thus preventing the second, stronger disulfide bond from forming and stabilizing the structure [104]. The great advantage of this compound is the oral route of administration. Single ascending doses between 1 mg and 800 mg were given to healthy participants, whereas multiple ascending doses from 30 mg do 800 mg were administered to healthy participants with Lp(a) values >30 mg/dL over the course of 14 days. Subjects were followed up for 105 days after the last administration. The average Lp(a) reduction was 63–65% at doses higher than 100 mg, which remained stable for the first month and progressively returned to baseline, with doses of 300 mg and 800 mg returning at two months. There was no significant change in other components of the lipid panel. Side effects were mild, ranging from headache to diarrhea, abdominal pain, nausea and fatigue [104].

Although Muvalaplin only transiently lowers Lp(a) values, it may represent a valid and cost-effective alternative to gene-interference based compounds given the oral route of administration [104].

**Fig. 3.**
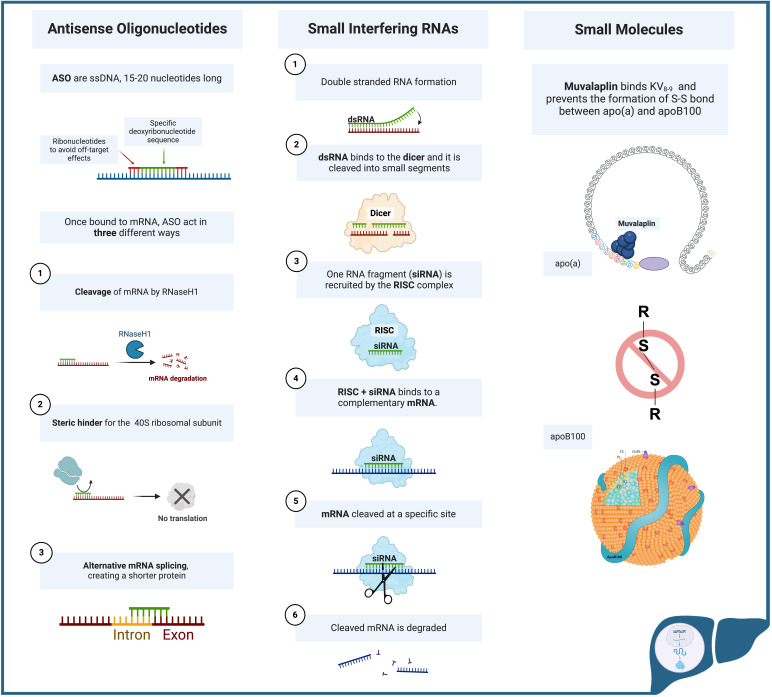
**Mechanisms of action of Antisense Oligonucleotides, Small Interfering RNAs and Small Molecules**. Apo(a), Apolipoprotein(a); ApoB-100, apolipoprotein B-100; ASO, antisense oligonucleotides; dsRNA double-stranded ribonucleic acid; mRNA, messenger ribonucleic acid; RISC, RNA-induced silencing complex; RNaseH1, ribonuclease H1; siRNA, small interfering ribonucleic acid; ssDNA, single-stranded deoxyribonucleic acid; KV: kringle V; S-S, disulfide bond. Created with BioRender.com.

A summary of novel therapeutic agents for Lp(a) and their mechanism of action is provided in Table 2 (Ref. [86, 87, 89, 97, 98, 101, 102, 103, 104]) and Fig. 3.

**Table 2.** **Principal characteristics of novel therapeutic compounds targeting Lp(a)**.

Medication	Clinical Trial Phase	Mechanism of Action	Route, Dosage and Frequency of Administration	%Lp(a) Reduction	Effect on LDL-c	References
Pelacarsen	Phase 3	ASO directed toward Apo(a) mRNA	SQ, 80 mg monthly	>90%	15–50% reduction	[86, 87, 89]
Olpasiran	Phase 3	siRNA directed toward Apo(a) mRNA	SQ, 75 mg or 225 mg every 12 weeks	70–90%	22–25% reduction	[97]
Zerlasiran	Phase 2	siRNA directed toward Apo(a) mRNA	SQ, dose and frequency to be determined	85–95%	13–26% reduction	[98, 101, 102]
Lepodisiran	Phase 2	siRNA directed toward Apo(a) mRNA	SQ, dose and frequency to be determined	96–98%	Not disclosed	[103]
Muvalaplin	Phase 2	Small molecule inhibitor preventing Apo(a)-ApoB-100 bond formation	PO, dose and frequency to be determined	63–65%	No change	[104]

ASO, antisense oligonucleotide; Apo(a), apolipoprotein(a); ApoB-100, apolipoprotein B-100; Lp(a), lipoprotein (a); mRNA, messenger RNA; PO, per os (by mouth); siRNA, small interfering RNA; SQ, subcutaneous.

## 7. Summary Remarks and Future Perspectives

Lp(a) bears independent risk of ASCVD and represents an underestimated but important biomarker for risk stratification in the general population. Despite the abundant evidence supporting universal screening, to date, such an indication is not explicitly endorsed by the AHA/ACC guidelines, which consider Lp(a) a risk enhancer [63]. This may reflect the fact that effective and etiologic therapies, though on the horizon, are not presently clinically available. More data from clinical trials are expected to provide more robust information.

When evaluating the lipid panel, the contribution of Lp(a)-c to the overall LDL-c is not adequately considered, sometimes leading to an overestimation of the calculated LDL-c and misclassification of patients with familial hypercholesterolemia [67].

Identifying the amount of cholesterol carried by each lipoprotein would not only risk stratify patients more accurately, but would also guide the therapeutic management based on the selectivity of each drug [44].

The focus of researchers has been directed toward gene transcription interference, which gives the possibility of selectively targeting *LPA* gene expression in hepatocytes. Selectivity is achieved by including GalNac in the nucleotide strands, which reduce any off-target effects.

Three major strategies have been pursued: antisense oligonucleotides, small interfering RNAs and small molecules. Potential future targeted therapies for Lp(a) are Pelacarsen, Olpasiran, Zerlasiran, Lepodisiran and Muvalaplin.

All developed compounds based on gene interference have shown a longer lasting effect on Lp(a) reduction compared to the limited available therapies [86, 87, 89, 97, 98, 101, 102, 103].

Administration of these drugs could be performed during follow up appointments without the need of receiving further home or hospital instruction for medication.

Currently phase III trials for both Pelacarsen and Olpasiran are ongoing, named Lp(a)HORIZON (NCT04023552) and OCEAN(a) (NCT05581303), respectively. The major difference between the two relates to the inclusion criteria. For Pelacarsen [105] a broader range of atherosclerotic diseases has been considered, whereas for the OCEAN(a) trial [106] includes subjects with previous MI or coronary revascularization with percutaneous coronary intervention (PCI) and at least one additional risk factor.

Both trials include as primary endpoints the time to cardiovascular death, MI, or urgent coronary revascularization. The Lp(a)HORIZON trial also adds non-fatal strokes. Lp(a)HORIZON excludes pregnant women, whereas the Olpasiran trial does not. The selection of the patient population included in these clinical trials will likely affect the indications approved by FDA. A subtle but relevant difference in the secondary endpoint is the inclusion of time to non-urgent coronary revascularization in the Olpasiran trial, which allows inclusion of patients with stable coronary disease.

A final consideration must be acknowledged on inclusion criteria based on Lp(a) values. For most of the trials, subjects were enrolled if Lp(a) was greater than 60 mg/dL or even more. The currently recruiting phase III trials do not include subjects that are in the previously mentioned “gray zone”, excluding subjects with Lp(a) levels of 75–125 nmol/L (30–50 mg/dL) who are still at increased cardiovascular risk [64].

Elucidation of the risk reduction in this category of patients will also be necessary to plan future preventive strategies for this important subgroup.

Moreover, no study is currently addressing the issue of prevention conferred by these medications for subjects with subclinical atherosclerotic disease, irrespective of the level of Lp(a) value.

Given that elevated Lp(a) values can potentially be detected relatively early in life, along with the fact that Lp(a) elevation has implications for first-degree relatives, a more targeted strategy for screening would be desirable.

Current FDA approved drugs, PCSK9-i monoclonal antibodies and Inclisiran are the only medications that can lower Lp(a) and can be safely administered at present. For these drugs, the Lp(a) lowering is a secondary mechanism, as these primarily lower LDL-c. Thus, Lp(a) lowering is not an overt indication approved by the FDA for prescription of the two classes of medications.

Since the reduction in Lp(a) levels is 20–30%, these medications may still represent an important treatment option for patients with dyslipidemia and moderately elevated Lp(a).

## 8. Conclusions

To date, no targeted therapy for Lp(a) lowering is approved by the FDA. Given the increased recognition to Lp(a) as a major initiator and propagator of cardiovascular disease, the novel etiologic therapies for this biomarker may eventually lead to improved screening strategies for primary and secondary prevention.

The current drugs being scrutinized in this review bring a great promise of change in the approach to cardiovascular disease risk reduction and treatment, and have the potential to decrease healthcare burden and significantly improve quality of life.
